# IoT Monitoring of Indoor Air Quality in Dairy Goat Barns: The Role of Building Characteristics and Litter Management

**DOI:** 10.3390/ani15223332

**Published:** 2025-11-19

**Authors:** Stefania Celozzi, Roberto Ambrosini, Luca Rapetti, Silvana Mattiello, Alberto Finzi

**Affiliations:** 1 Department of Agricultural and Environmental Sciences, University of Milan, Via Celoria 2, 20133 Milano, Italy; stefania.celozzi@unimi.it (S.C.); luca.rapetti@unimi.it (L.R.); silvana.mattiello@unimi.it (S.M.); 2 Department of Environmental Science and Policy, University of Milan, Via Celoria 26, 20133 Milano, Italy; roberto.ambrosini@unimi.it; 3 Centre of Applied Studies for the Sustainable Management and Protection of Mountain Areas (CRC Ge.S.Di.Mont.), University of Milan, Via Morino 8, 25048 Edolo, Italy

**Keywords:** low-cost sensors, real-time monitoring, animal welfare, dairy goat housing

## Abstract

Air quality in livestock housings impacts animal welfare; however, information on air quality in dairy goat barns is still limited. The air quality and environmental conditions of two farms in northern Italy were monitored for seven days after litter renewal in both summer and winter. The farms had different barn designs and litter replacement frequencies. Internet of Things (IoT) sensors were used to measure CO_2_, NH_3_, PM_2.5_, temperature and humidity. Results suggest that building structure, particularly the management of openings, along with litter replacement frequency, and season, significantly affect gas concentrations, while PM_2.5_ seems to be more related to the external conditions. The recommended thresholds for goat health and welfare were all met (537 vs. 778 ppm for CO_2_, 1.78 vs. 3.29 ppm for NH_3_ and 3.4 vs. 12.7 µg m^−3^ for PM_2.5_ in Farms A and B, respectively). The low average temperature humidity index values recorded in winter at Farm A (45.9) suggest potential cold stress, which could be mitigated through improved barn opening management. Our research introduces a novel use of IoT sensors in the frame of precision livestock farming to monitor air quality in goat barns, allowing data-driven interventions to improve animal welfare.

## Simple Summary

Although the air quality inside barns plays a significant role in animal welfare, there is still limited information available regarding dairy goat barns. To fill this knowledge gap, we studied two goat farms in northern Italy in both summer and winter. The farms had different types of buildings and cleaned the bedding at different frequencies. We used smart sensors, known as Internet of Things (IoT) devices, to measure air quality by tracking carbon dioxide, ammonia, particulate matter, temperature and humidity. Our findings revealed that the design of the barns, particularly the management of openings such as windows, along with the frequency of bedding changes, and the season, had a significant impact on air quality parameters. On both farms, air quality remained within the recommended levels for the health and comfort of the goats. However, temperatures were quite low on one farm during winter, which could have stressed the animals. This study demonstrates the impact of different factors on environmental conditions within goat barns and shows how smart farming technology can assist farmers in monitoring these conditions, thereby improving the animals’ care and comfort.

## 1. Introduction

In the European dairy goat sector, intensive and semi-intensive farming systems have become widespread, especially for highly productive breeds [1]. At the same time, increased consumer awareness of animal welfare and the need to adapt to EU regulations on the hygiene of food of animal origin have led to the adoption of farming techniques that take these aspects into account [1].

Animal welfare is negatively affected by poor air quality, e.g., when high concentrations of certain hazardous pollutants are present, because these substances are generally recognized as a threat to health and safety or as a stress factor. In farm environments, the most studied gaseous pollutants affecting animal welfare are NH_3_, CO_2_, and H_2_S, but also particulate matter such as PM_2.5_–PM_10_ [2]. High concentrations of NH_3,_ in particular, have been shown to significantly reduce locomotor and feeding behavior [3,4] and are often considered indicators of a pathological condition. They are also associated with respiratory problems and reduced weight gain [4]. Adverse environmental conditions can also compromise animal welfare: for example, Temperature Humidity Index (THI) values above 70 increase the risk of heat stress, while at THI values below 55, goats begin to suffer from cold stress [5].

The air quality and ambient conditions in barns are influenced by multiple factors, including building characteristics, ventilation rate, litter management, animal species, age, diet, stocking density and activity level [2]. In Italy, dairy goats are commonly housed in naturally ventilated barns that rely on natural airflow rather than mechanical systems, causing ventilation rates to fluctuate with building orientation, structural design, external weather conditions, and the time of day and season [2,6]. Such variability can lead to elevated gas concentrations, with direct implications for goat welfare.

Air pollutant monitoring in livestock buildings has traditionally relied on advanced techniques, such as photoacoustic spectroscopy (PAS), Fourier transform infrared spectroscopy (FTIR), tunable diode laser absorption (TDLA) spectroscopy, and chemiluminescence analyzers [7,8,9,10]. While highly accurate, these instruments are expensive, labor-intensive, and unsuitable for long-term use in harsh environments, which limits their use in an agricultural context [11]. Recently, simpler sensor-based systems (e.g., electrochemical sensors) have gained traction due to their capacity for continuous measurements, lower costs and ease of use [12,13,14,15,16] and are increasingly applied within Precision Livestock Farming (PLF) frameworks [17]. Although extensive research on indoor air quality monitoring has been conducted for pigs, cattle, and poultry [17,18,19,20], as well as studies conducted on large panels of farms [21], evidence for the dairy goat sector remains limited, with only a few studies published to date [22,23,24].The aim of this study is to evaluate air quality in goat farms as a function of management and environmental factors: (i) building characteristics and management, (ii) season and time of day, and (iii) litter renewal frequency. Additionally, the study explored the innovative use of low-cost and easy-to-use devices for continuous monitoring of air quality and environmental conditions in dairy goat barns.

## 2. Materials and Methods

### 2.1. The Monitored Barns

**Figure A1:**
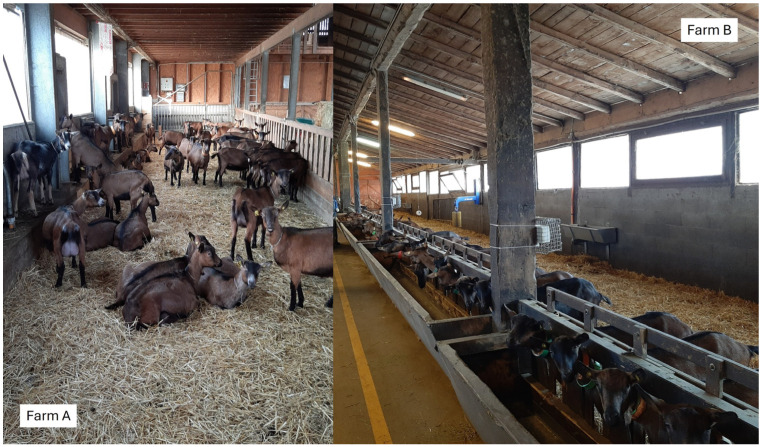
Images of the two monitored farms.

The monitored Farms (A and B) were located in the Lombardy Region (Northern Italy) and raised Alpine dairy goats housed on deep litter (Figure A1). Average weather conditions in winter and summer are reported in Table 1. The housing area was 20–25 m long on both farms.

The farms differed in flock sizes, building design and litter management (Table 1). At both farms, stocking density and airspace complied with the recommended thresholds of 1.5–2 m^2^ head^−1^ and 7 m^3^ head^−1^, respectively [1]. The air inlet surface (barn windows and gates surfaces) was greater in summer on both farms, due to more open windows and gates, and in both seasons, this parameter was higher on Farm A. The air outlet surface (the upper roof opening surface) was also higher on Farm A; the slight seasonal variation was only due to the different number of goats on this farm. The farms also differed in litter management, particularly in the frequency of renewal (Table 1). On Farm A, litter was renewed more frequently in summer than in winter. Higher water intake by the animals leads to faster soiling of the litter, and fly and parasite counts were greater during the warm season [25].

On Farm A, during the winter, the goats were pregnant and non-lactating; the daily individual amount of the administered diet was about 2.15 kg as fed (1.88 kg dry matter, DM), consisting of meadow hay (74% of total DM), lucerne hay (pelleted), and steam-rolled barley and maize. The dietary crude protein (CP) was 12% DM. During the summer, the goats were in lactation and were fed a diet (2.5 kg DM) with a lower forage-to-concentrate ratio (58%) and a lower CP content (10.5% on DM). On Farm B, both in winter and summer, the goats were lactating and were fed a diet (3.6 kg DM) with a forage-to-concentrate ratio of about 60%, consisting of meadow hay, alfalfa hay and a commercial concentrate mix. The dietary crude protein (CP) was 16.6% DM. The individual milk yield (kg d^−1^) and milk urea content (mg dL^−1^) on Farm A (summer) were, on average, 2.3 and 33.7; on Farm B, the values recorded were 3.3 and 30.1 and 4.0 and 37.0 during winter and summer, respectively.

### 2.2. Monitoring Units

Monitoring was carried out in 2023 in two different seasons (winter and summer) by installing two monitoring units inside the barns. In both seasons, monitoring was carried out for seven days after litter renewal. In addition, on Farm A, monitoring was repeated when litter was 60- (summer) or 90 day-old (winter). The monitoring units were protected by a steel cage and positioned 7–8 m apart at a height of 1.3 m. The monitoring units (N11, IBT Systems, Milano, Italy) were powered by a 12 V battery (lasting for about 20 days) and sent data every 10 s to a gateway, which averaged data every 10 min and sent it to a cloud. Recorded data was available in real time via a dashboard and smartphone application. Temperature, relative humidity, particulate matter (PM_2.5_ and PM_10_), NH_3_, H_2_S and CO_2_ sensors were installed on the monitoring units. The sensor’s characteristics are shown in Table 2. A detailed description of the technology of communication, energy harvesting, connection between parts, validation and calibration procedure, and cost of the system is available in [26].

In addition, outdoor temperature and relative humidity were obtained by the meteorological station closest to the farms (700 m from Farm A and 1870 m from Farm B), while outdoor PM_2.5_ concentration was obtained only by the meteorological station close to Farm A. These meteorological stations were part of the network of the Regional Agency for Environmental Protection of Lombardy (ARPAL).

### 2.3. Data Processing and Statistical Analyss

THI was calculated according to the equation developed by [27]:

(1) $$THI=\left(1.8\;\times \;T+32\right)-[\left(0.55-0.0055\;\times \;RH\right)\times \left(1.8\;\times \;T-26.8\right)]$$

where *T* = air temperature (C°), *RH* = relative humidity (%). Both parameters were measured by the monitoring units installed inside the barns.

Because the mean concentrations of CO_2_, NH_3_, PM_2.5_ and THI were temporally autocorrelated and hierarchically structured as they represented repeated measures within the same day and farm, they were analyzed with linear mixed-effects models (LMMs). Three different model types were used depending on the experimental design and the comparison of interest. The first set of models compared air quality parameters between the two farms during periods when the litter was fresh, i.e., on the day of litter renewal and in the following 6 days. The general model structure was:

*response* ~ *Farm* + sin(*hour*) × cos(*hour*) + (1 | *Day*)

Here, *Farm* was included as a two-level fixed effect, while *Day* was treated as a random factor to account for repeated measurements within days. The sine and cosine transformations of time and their interaction epresented the first Fourier term, allowing the model to capture circadian (24 h) cycles in gas concentrations [28].

Temporal autocorrelation among residuals within each day was modeled using a first-order autoregressive structure [corAR(1)], and potential heteroscedasticity between farms was accounted for by specifying a varIdent variance structure.

The second set of models was fitted to evaluate possible differences in air quality at different times of the day; moments characterized by opposite conditions were considered: periods of high animal activity and opening of the barn, identified during the daytime, and periods of low or no animal activity and closing of the barn, identified during the nighttime (i.e., daytime = 10 a.m.–2 p.m. and nighttime = 10 p.m.–2 a.m.). In both seasons, the average percentage of active animals (estimated by observing the animals using a scan sampling technique at 15 min scan intervals) was 50% during daytime and 29.5% during nighttime.

These models were fitted separately for each farm, and their general structure was

*response* ~ *Period* × *Season* + sin(*hour*) × cos(*hour*) + (1 | *Day*/*Period*)

where *Period* and *Season* are dichotomous variables accounting for day vs. night and summer vs. winter, respectively. Their interaction was also included to account for differential effects of the period in different seasons. Random intercepts were included for *Day* nested within *Period*. Autocorrelation within *Day*/*Period* was modeled using a corAR(1) process, while heterogeneity of residual variance between combinations of *Season* and *Period* was handled using a varIdent variance structure.

Finally, on Farm A, where litter renewal was performed every 60–90 days, the effect of litter age on the concentration of gases and PM_2.5_ was analyzed in a third set of models with a general structure:

*response* ~ *litter_condition* + sin(*hour*) × cos(*hour*) + (1 | *Days_since_litter_change*)

Here, *Days_since_litter_change* represents the number of days elapsed since the last litter replacement, while *litter_condition* is a categorical variable grouping consecutive days into two levels (fresh vs. aged litter). As in the other models, we modeled temporal autocorrelation with a corAR(1) structure and accounted for potential differences in residual variance among levels of *litter_condition* using a varIdent function.

For each dependent variable and model type, we tested the significance of fixed effects using marginal ANOVA (type II sums of squares). Model assumptions were verified by inspecting residuals for homoscedasticity, normality, and absence of patterns across time.

All statistical analyses were performed in R version 4.3.2 [29] using the package nlme [30]. All results are presented as model estimates (mean ± SE) derived from the final fitted models.

## 3. Results

### 3.1. Air Quality and Ambient Conditions During the First Seven Days After Litter Renewal

**Figure 1:**
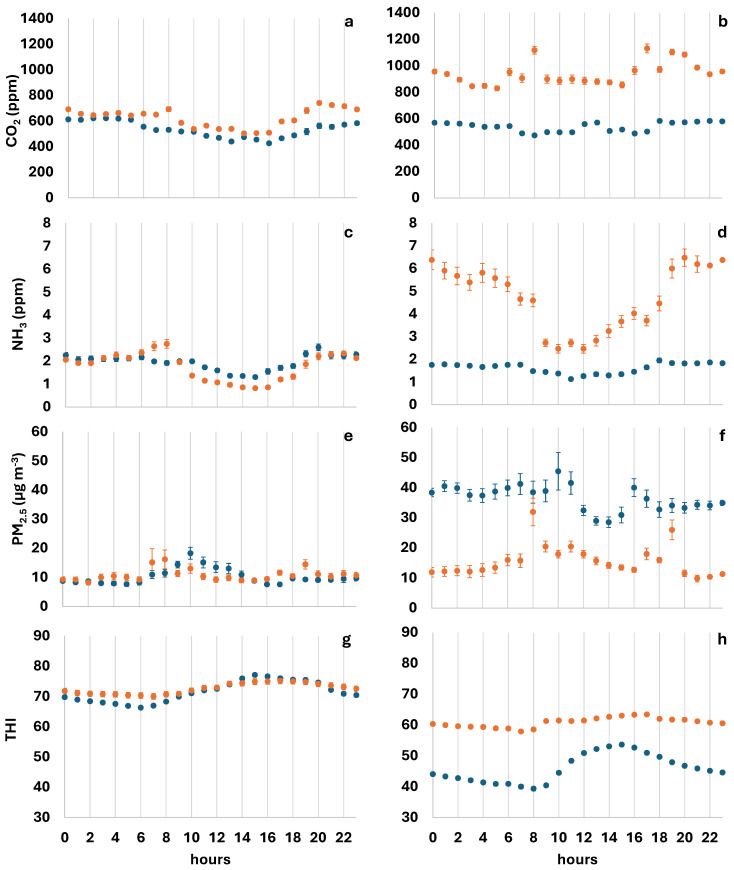
Data recorded on Farm A (blue dot) and Farm B (orange dot), presented as an hourly average and standard error, over a 24 h period: CO_2_ in summer (**a**) and winter (**b**); NH_3_ in summer (**c**) and winter (**d**); PM_2.5_ in summer (**e**) and winter (**f**); THI in summer (**g**) and winter (**h**).

A descriptive presentation of the monitored parameters is shown in Figure 1 as an hourly average over a 24 h period of the seven days after litter renewal.

**Figure A2:**
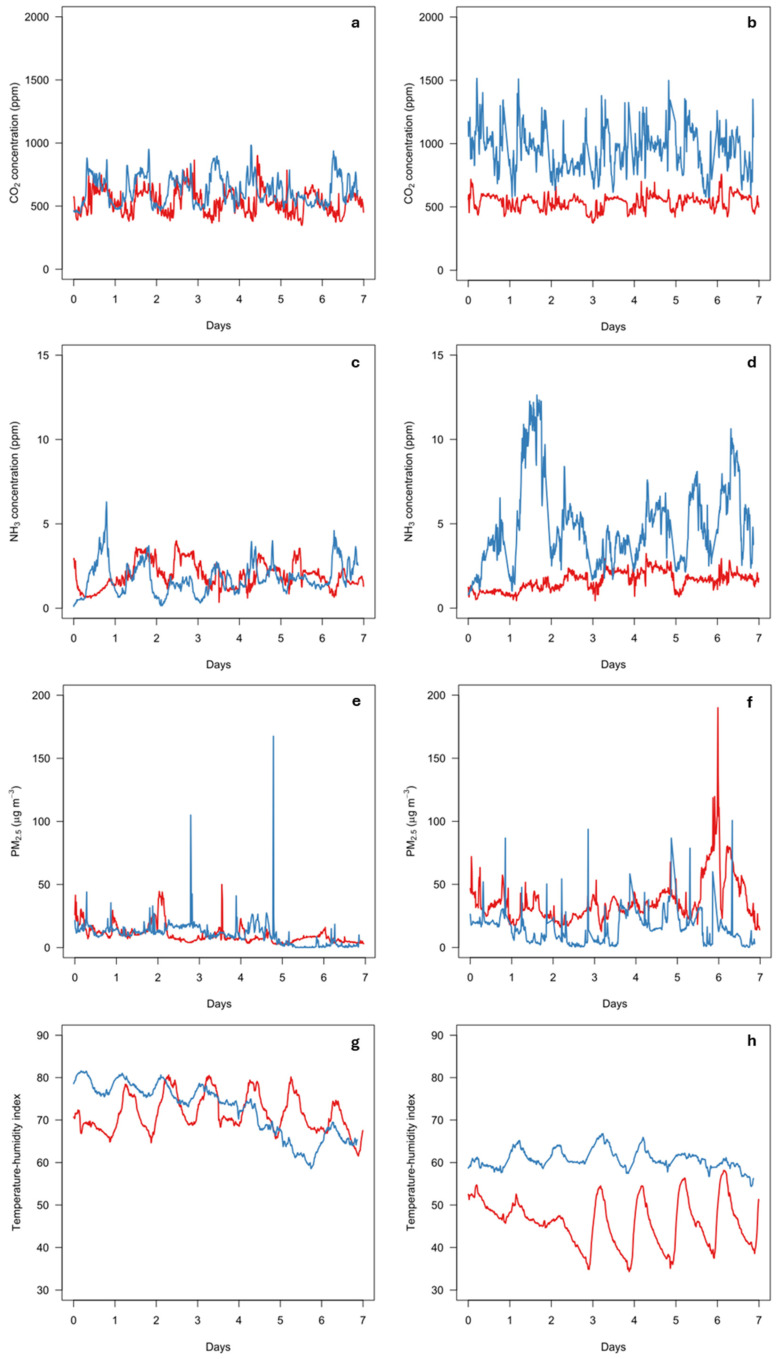
Parameters recorded on Farm A (red line) and Farm B (blue line): CO_2_ trends during summer (**a**) and winter (**b**); NH_3_ trends during summer (**c**) and winter (**d**); PM_2.5_ trends during summer (**e**) and winter (**f**); THI trends during summer (**g**) and winter (**h**).

The average CO_2_ concentrations during the 6 days after litter renewal were similar in the two farms during summer (monitoring period from June to August 2023; mean ± SE: 534.7 ± 2.98 ppm and 625.8 ± 3.64 ppm on Farms A and B, respectively; Figure 1a), whereas higher CO_2_ concentrations were observed on Farm B during the winter season (monitoring period from January to March 2023; mean ± SE: 538.5 ± 1.78 ppm and 946.5 ± 5.70 ppm on Farms A and B, respectively; Figure 1b). The average values of NH_3_ were also similar at the two farms in summer (mean ± SE: 1.95 ± 0.02 ppm and 1.78 ± 0.03 ppm on Farms A and B, respectively; Figure 1c), but showed large differences in winter (mean ± SE: 1.61 ± 0.02 ppm and 4.97 ± 0.08 ppm on Farms A and B, respectively; Figure 1d), especially at Farm B, where NH_3_ concentrations reached a maximum value of 12.6 ppm (Figure A1). PM_2.5_ concentrations were lower in summer than in winter on both Farms in the first 6 days after litter renewal (mean ± SE 10.2 ± 0.28 µg m^−3^ vs. 36.6 ± 0.68 µg m^−3^ at Farm A and 10.8 ± 0.39 µg m^−3^ vs. 14.9 ± 0.52 µg m^−3^ at Farm B in summer and winter, respectively; Figure 1e,f). In summer, PM_2.5_ concentrations were similar on both farms, and the trends were relatively constant, except for two records at Farm B (maximum PM_2.5_ concentration = 167.5 µg m^−3^, as shown in Figure A2). The average external concentration of particulate matter was 8 µg m^−3^ at Farm A in summer. In winter, the worst conditions were recorded at Farm A, with a maximum PM_2.5_ concentration of 190 µg m^−3^ (Figure A2). In this season, an external PM_2.5_ concentration of 31 µg m^−3^ was recorded at this farm.

During summer, the THI was relatively constant at Farm A, whereas at Farm B it decreased with increasing litter age due to the meteorological conditions of the monitored days (Figure 1g). The mean summer THI values (mean ± SE: 71.5 ± 0.14 and 72.6 ± 0.19 at Farms A and B, respectively) were similar between the two farms, as were the maximum THI values recorded in this season (80.6 and 81.5 at Farms A and B, respectively, as shown in Figure A2). The average THI values were similar to outdoor values, measuring 71 on both farms. In winter, the average trend of THI was very different on the two farms: 45.9 ± 0.17 and 60.8 ± 0.08 (mean ± SE on Farms A and B, respectively; Figure 1h), remaining more constant and reaching higher values at Farm B and showing large variations and much lower values at Farm A. In particular, in the latter farm, critical environmental conditions were recorded (i.e., minimum THI value = 34.3 as shown in Figure A2), suggesting a probable cold stress situation. The average indoor THI at Farm A closely matched the outdoor value at 45. In contrast, Farm B had a higher indoor THI than the outdoor average, measuring 53. H_2_S was always around 0–0.1 ppm, well below the suggested critical threshold for H_2_S, which is set at 2.5 ppm [1]; however, during litter renewal, H_2_S concentrations between 0.7 and 2.1 ppm were recorded for short periods (10–20 min) but in the absence of animals in the barn.

### 3.2. Comparison Between Farms When the Litter Was of the Same Age

**Figure 2:**
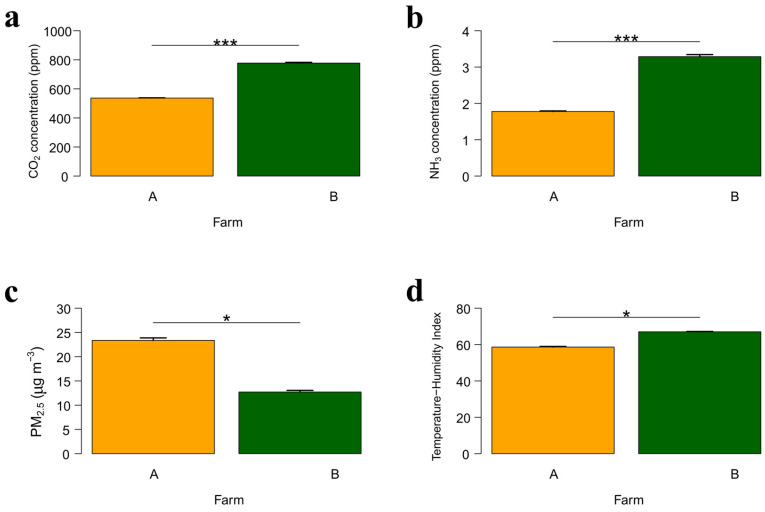
Average gas and particulate matter concentrations and THI on Farms A and B within 6 days after the litter renewal: CO_2_ (**a**); NH_3_ (**b**); PM_2.5_ (**c**); THI (**d**). Asterisks denote groups that differ significantly (* = *p* < 0.05; *** = *p* < 0.001).

For all the variables analyzed, their interaction between Sin(*hour*) and Cos(*hour*) had significant effects (Table 3), confirming a pronounced diurnal cycle. CO_2_ and NH_3_ concentrations were significantly higher on Farm B than on Farm A (+246.45 ± 40.39 ppm; Table 3 and Figure 2a, and +1.73 ± 0.40 ppm; Table 3 and Figure 2b, respectively), whereas PM_2.5_ showed lower average values on Farm B (−10.45 ± 4.64 µg m^−3^; Table 3 and Figure 2c). In contrast, THI exhibited higher mean values on Farm B (+8.31 ± 3.65; Table 3 and Figure 2d). The intra-daily temporal correlation coefficients (φ) estimated through the AR(1) structure ranged from 0.74 for PM_2.5_ to 0.9996 for THI, indicating strong temporal autocorrelation, particularly for the microclimatic variables. Residual variances differed between farms, with greater variability observed on Farm B for CO_2_, NH_3_, and PM_2.5_. Conversely, THI showed slightly lower residual variance on Farm B compared with Farm A. Overall, the models revealed clear farm-specific differences and consistent diurnal patterns across all variables, suggesting coherent temporal dynamics between gaseous emissions and environmental conditions.

### 3.3. Effect of Season and Time of Day Within Farm

**Figure 3:**
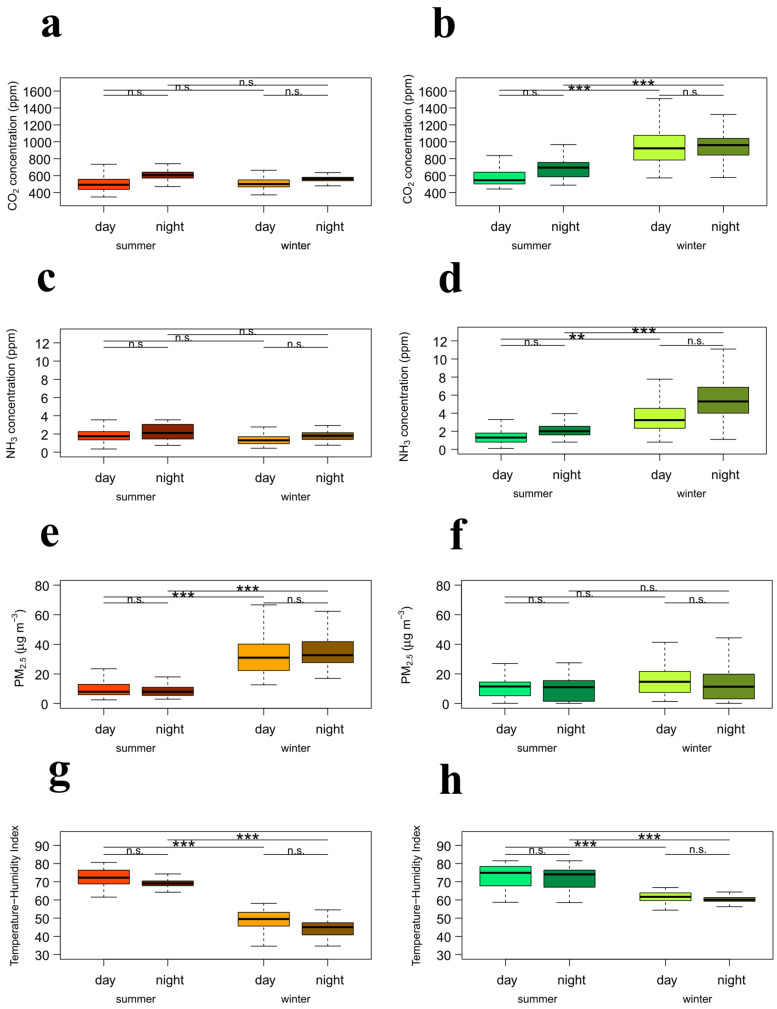
Effects of season and period of day (day = 10 a.m.–2 p.m.; night = 10 p.m.–2 a.m.) on CO_2_ at Farm A (**a**) and B (**b**); NH_3_ at Farm A (**c**) and B (**d**); PM_2.5_ at Farm A (**e**) and B (**f**); THI at Farm A (**g**) and B (**h**). Asterisks denote groups that differ significantly (n.s. = *p* ≥ 0.05; ** = *p* < 0.01; *** = *p* < 0.001).

Both farms exhibited a clear sinusoidal trend of all variables throughout the day (Table 4). For CO_2_, significant seasonal effects were observed on Farm B where winter concentrations were markedly higher than those recorded in summer, both during the day and at night (Table 4, Figure 3b). On Farm A, CO_2_ did not vary significantly between periods of the day in both seasons (Table 4, Figure 3a). No significant period–by season interaction effects were detected, indicating that there was no day-night difference in CO_2_ concentrations in both seasons (Table 4, Figure 3a). On Farm B, NH_3_ concentrations were significantly higher in winter than in summer, both during the day and at night (Table 4, Figure 3d). The period by season interaction indicated a not significant day–night difference in NH_3_ concentrations in both seasons. On Farm A, neither seasonal nor period effects were significant (Table 4, Figure 3c). PM_2.5_ concentrations were significantly higher in winter than in summer on Farm A, both during the day and at night (Table 4, Figure 3e). On Farm B, seasonal and period effects were not significant (Table 4, Figure 3f). For THI, both farms showed highly significant seasonal effects (Table 4, Figure 3g,h), with lower values in winter than in summer (Table 4). Regarding the period by season interaction no significant interaction was observed in both Farms.

### 3.4. Effect of Litter Age Within Season

**Figure 4:**
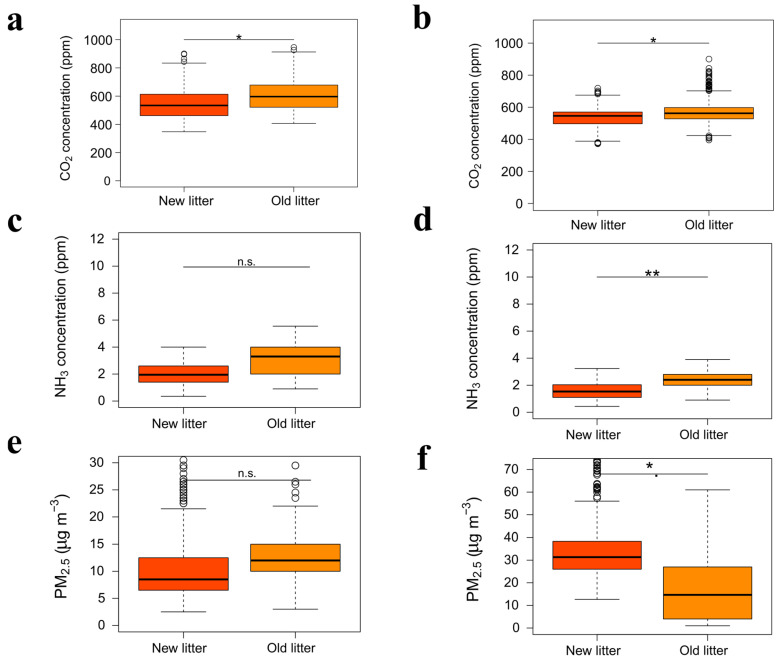
The indoor air quality and ambient conditions on Farm A with new and old litter. Summer season: CO_2_ (**a**), NH_3_ (**c**), PM_2.5_ (**e**); winter season: CO_2_ (**b**), NH_3_ (**d**), PM_2.5_ (**f**). Asterisks denote groups that differ significantly (n.s. = *p* ≥ 0.05; * = *p* < 0.05; ** = *p* < 0.01).

In summer, CO_2_ concentrations were significantly higher with the old litter than with the new one (Table 5, Figure 4a), with clear daily oscillations associated with both sine and cosine terms, indicating pronounced daily cycles in CO_2_ emissions (Table 5). NH_3_ concentrations tended to increase with the age of the litter (Table 5, Figure 4c) and showed a significant sinusoidal daily pattern (Table 5). Conversely, PM_2.5_ was not significantly affected by the age of the litter (Table 5, Figure 4e). In winter, CO_2_ and NH_3_ concentrations were significantly influenced by the age of the litter (Table 5, Figure 4b,d), with higher levels recorded at the end of the period. For PM_2.5_, concentrations were significantly lower at the end of the period (Table 5, Figure 4f), while no significant daily patterns were observed.

## 4. Discussion

### 4.1. THI

Although the mean THI values of both farms remained within the thermoneutral range of goats (i.e., between 55 and 70 [5]), the maximum summer values showed a critical condition predisposing to heat stress. in winter, critical environmental conditions were recorded on Farm A (i.e., minimum THI value = 34.3 as shown in Figure A1), suggesting a probable cold stress situation.

Both monitored farms were naturally ventilated. The indoor environmental conditions revealed the importance of building design and the management of air inlet and outlet surfaces (i.e., openings). These factors played a crucial role in regulating ventilation and influencing the THI. Monitoring of environmental conditions revealed very low THI values on Farm A in winter, probably due to spacious openings (i.e., air inlet surface; Table 2). The average THI value here was similar to the outdoor level and remained below 55, suggesting that the goats experienced low thermal comfortbecause cold stress can occur below this threshold [5]. In contrast, on Farm B, the winter openings were smaller, and this may have contributed to making the THI average value almost ten points higher than the outside one and above the threshold proposed by [5]. On the other hand, in summer the THI values of Farms A and B were more similar to each other and closely matched outdoor levels. This similarity between the indoor and outdoor conditions of the two farms was probably due to the greater openness of the windows and doors of the barns, which is typical of the summer period. The average THI values for this season are acceptable when considering a threshold of 70 for heat stress, while the maximum values above 80 recorded on both farms indicate potentially critical conditions, as heat stress is considered severe when the THI exceeds 75 [5]. However, since these high THI values occurred only for short periods, they are unlikely to pose a major concern, though monitoring animals’ clinical responses to THI fluctuations remains important. In the literature, effects of heat stress have been reported in Alpine goats, which decreased milk production after exposure to a THI of 79 for five weeks, and in Saanen goats, where a decrease in milk yield of 3% and 13% was recorded when exposed to a THI of 81 and 89 for four days, respectively [31].

### 4.2. NH_3_ and CO_2_

On both farms, CO_2_ concentrations remained below the recommended threshold of 2500 ppm for goats [1], while NH_3_ concentrations, with the exception of a few isolated exceedances in farm B, were generally maintained below the recommended threshold for goats of 10 ppm [1]. Furthermore, the concentrations of NH_3_ and CO_2_ never exceeded the allowed threshold established by the Italian law n.81/2008 about human protection and safety in the workplace.

The effect of barn opening management on THI also applies to indoor air quality, particularly NH_3_ and CO_2_ concentrations. Overall, Farm B had poorer air quality than Farm A, likely due to its smaller air inlet and outlet openings. Seasonal and daily air quality analyses revealed that Farm A had lower NH_3_ and CO_2_ concentrations in winter than in summer; however, these differences were not statistically significant. While increased winter ventilation improved air quality, it may have been excessive, given the very low THI. In contrast, Farm B had higher NH_3_ and CO_2_ levels in winter due to restricted airflow from limited door and window openings. Slightly higher concentrations of CO_2_ and NH_3_ were detected in both farms during the night than during the day, although these differences were not significant. The air quality observed at Farm B aligns with findings by [23], who reported higher NH_3_ and CO_2_ concentrations in winter and lower levels in summer due to reduced winter ventilation (0.4–0.8 m^3^ h^−1^ per goat) compared to summer (12–18 m^3^ h^−1^ per goat). Their study recorded NH_3_ concentrations of 2.8–7.4 ppm in winter and 1.0–3.4 ppm in summer, while CO_2_ levels ranged from 1988 to 2354 ppm in winter and from 754 to 857 ppm in summer in barns with solid and slatted floors, respectively. Despite the well-established positive correlation between milk urea level (MUL) and urinary nitrogen (UN) excretion [32], our study, particularly for Farm B, suggests that barn opening management has a greater influence on ammonia concentration than UN excretion. In ruminants, urinary urea nitrogen is a major nitrogen excretion product. After being excreted in urine, urea is rapidly converted into ammonia by the enzyme urease, which is present in feces and soil, and subsequently lost to the atmosphere. The concentration of milk urea is an indicator of the amount of N excreted in urine, making it a useful tool to estimate urinary urea N and, consequently, potential ammonia emissions from livestock farms. Contrary to expectations, based on lower MUL in winter, ammonia concentrations were lower when MUL was higher.

The age of the litter significantly influenced indoor air quality, with both NH_3_ and CO_2_ concentrations being higher in both seasons when the litter was older than when it had just been renewed. These findings align with studies by [8,33] on NH_3_ and greenhouse gas emissions in pig barns reared on litter. In livestock buildings, CO_2_ production derives not only from animal respiration but also from manure [34]. When manure is frequently removed, its contribution to CO_2_ emissions is minimal. The authors of [35] estimated that in barns where manure is stored for less than three weeks, it accounts for only 10% of CO_2_ emissions. However, in goat barns with longer bedding renewal periods (4–5 weeks to 3 months), manure is responsible for 56% of CO_2_ emissions [36].

### 4.3. PM_2.5_

The literature does not provide any specific values for the recommended PM thresholds for goats, even if we can consider a range of 3–10 mg m^−3^ for animals housed as an acceptable threshold [18]. The concentrations of PM_2.5_ never exceeded the allowed threshold established by [37] to preserve human health.

PM_2.5_ levels in the barns seemed to be strongly influenced by external environmental conditions. On Farm A, higher concentrations were recorded in winter due to poorer outdoor air quality, while summer levels were lower and closely matched outdoor values. As previously mentioned, an assessment was not possible for Farm B due to the absence of nearby ARPA meteorological monitoring stations. Sevi et al. (2003) [6] reported respirable dust concentrations (2–5 µm particles) of 150 µg m^−3^ in sheep barns with 0–14-day-old litter, significantly higher than the levels recorded in the present study. Regarding the influence of the age of the litter on indoor air quality, in winter, PM_2.5_ levels tended to be higher when the litter was newer, whereas in summer, the opposite trend was observed. However, the differences in PM_2.5_ concentrations between new and old litter in summer had a lower significance than the differences in the winter period. In winter, the higher levels of PM_2.5_ detected in new litter are probably caused by the dustiness of fresh straw, which becomes damp over time. This reduces the amount of PM_2.5_ released into the air. In summer, when it is hotter, the litter may remain drier and therefore stay dustier. Sevi et al. (2003) [6] found that respirable dust levels in sheep farms increased with litter age, rising from 0.15 mg m^−3^ at 0–14 days to 0.95 mg m^−3^ at 43–56 days. However, since Sevi et al. (2003) [6] did not specify the season of measurement, direct comparisons with our results are difficult.

### 4.4. Limitations

Monitoring of both barns was only carried out during certain periods: seven days after litter replacement in two seasons and a few days before litter removal at Farm A. Monitoring over the course of a whole year would enable the entire life cycle of the bedding in the various repeating cycles throughout the year to be observed, thus providing a comprehensive overview of air quality in dairy goat barns under more heterogeneous conditions. However, this more in-depth monitoring was not possible due to limitations of the measurement system, as highlighted by [38], such as fouling of the monitoring units and the lifespan of the electrochemical sensors and batteries. The operating principle of electrochemical sensors involves an electrolyte that is consumed during the measurement process, which affects the lifespan of the sensor proportionally to the concentrations detected [7,39]. Based on the results obtained, we do not believe that the monitored goat barns presented conditions that compromised the sensors’ measurement accuracy for the entire monitoring period [40]. Future studies should allow for optimization of the measuring system’s management, either by prolonging the battery life or by substituting it with AC current. The latter option would, however, require work on the barn’s electrical system. Finally, extending the life of the sensors by reducing continuous monitoring to high-frequency monitoring could be achieved by sampling the internal air and sending it to the sensors.

## 5. Conclusions

The IoT-based sensor system used in the present study enabled continuous, non-invasive, real-time monitoring of environmental conditions and air quality in goat barns, marking the first reported application of a Precision Livestock Farming (PLF) tool for this purpose. Beyond demonstrating technical feasibility, the findings highlight how barn structure, the management of openings, and litter renewal frequency influence gas concentrations and overall air quality. While measured values were generally within recommended thresholds for animal health and welfare, instances of both heat and cold stress were detected, underlining the need for artificial ventilation and barn openings management. This preliminary study confirms the potential of IoT systems as practical tools for environmental monitoring in goat farming and points to future work extending to additional farms, seasons, and gases such as CH_4_.

## Figures and Tables

**Table 1:** Farm characteristics.

Parameter	Farm A		Farm B	
	Winter	Summer	Winter	Summer
Wheater conditions				
Temperature (°C)	5.9 ± 3.2	22.6 ± 3.8	11.5 ± 3.4	25.2 ± 7.3
Humidity (%)	73.1 ± 16.7	85.9 ± 15.0	61.0 ± 23.6	54.0 ± 25.3
Precipitation (mm)	0.0 ± 0.0	0.4 ± 2.3	0.0 ± 0.2	0.3 ± 1.3
Altitude (m a.s.l.)	300		600	
Barn orientation	E-W		E-W	
Building material	Wood (roof); Concrete-wood (walls)		Wood (roof); Concrete (walls)	
Goats (n.)	120	110	66	59
Stocking density (m^2^ head^−1^)	1.54	1.68	1.73	1.93
Airspace (m^3^ head^−1^)	14.3	15.6	9.8	11
Air inlet surface (m^2^ head^−1^)	0.61	0.67	0.25	0.43
Air outlet surface (m^2^ head^−1^)	0.38	0.41	0.07	0.08
Litter renewal frequency (d)	90	60	7	

**Table 2:** Characteristics of the sensors installed on the monitoring units. Manufacturers (*) SGX sensortech, Corcelles-Cormondreche, Switzerland; (**) Sensirion, Stäfa, Switzerland.

Parameter	Sensor	Type	Range	Accuracy
NH_3_	4NH3-100 (*)	Electrochemical	0–100 ppm	±10%
CO_2_	SCD30 (**)	Nondispersive infrared (NDIR)	400–10,000 ppm	30 ppm
H_2_S	4H2S-100 (*)	Electrochemical	0–100 ppm	±2%
Temperature	SHT3x/SHT4x (**)	CMOSens	−40–+125 °C	±0.1 °C
Humidity	SHT3x/SHT4x (**)	CMOSens	0–100%	±2%
PM	SPS30 (**)	Laser scattering	0–1000 µg m^−3^	±10 µg m^−3^ (PM_2.5_) ±25 µg m^−3^ (PM_10_)

**Table 3:** Fixed effects of the linear mixed-effects models (LMMs) for the mean concentrations of CO_2_, NH_3_, PM_2.5_ and THI on Farms A and B. φ is the temporal autocorrelation coefficient estimated by the model.

Variable	Effect	Estimate	SE	DF	t	*p*
CO_2_	φ	0.89	-	-	-	-
	Intercept	536.50	27.32	3852	19.64	<0.001
	Farm B	246.45	40.39	30	6.10	<0.001
	sin(hour)	27.94	7.89	3852	3.54	<0.001
	cos(hour)	55.47	7.07	3852	7.84	<0.001
	sin(hour) × cos(hour)	−42.38	12.86	3852	−3.30	0.001
NH_3_	Φ	0.98	-	-	-	-
	Intercept	1.63	0.17	3852	9.79	<0.001
	Farm B	1.73	0.40	30	4.26	<0.001
	sin(hour)	0.45	0.10	3852	4.29	<0.001
	cos(hour)	0.59	0.10	3852	5.82	<0.001
	sin(hour) × cos(hour)	−0.64	0.12	3852	−5.47	<0.001
PM_2.5_	Φ	0.74	-	-	-	-
	Intercept	23.10	3.23	2552	7.16	<0.001
	Farm B	−10.45	4.64	30	−2.25	0.032
	sin(hour)	2.92	0.69	2552	4.26	<0.001
	cos(hour)	−0.94	0.63	2552	−1.50	0.135
	sin(hour) × cos(hour)	−3.50	1.23	2552	−2.85	0.004
THI	Φ	0.99	-	-	-	-
	Intercept	58.21	2.99	3852	19.50	<0.001
	Farm B	8.31	3.65	30	2.28	0.03
	sin(hour)	−2.36	0.12	3852	−20.09	<0.001
	cos(hour)	−0.45	0.14	3852	−3.21	0.001
	sin(hour) × cos(hour)	1.02	0.14	3852	7.43	<0.001

**Table 4:** Fixed effects of the linear mixed-effects models (LMMs) for the mean concentrations of CO_2_, NH_3_, PM_2.5_ and THI.

Variable	Farm	Effect	Estimate	SE	DF	t	*p*
CO_2_	A	φ	0.91	-	-	-	-
		Intercept	529.91	16.63	1975	31.86	<0.001
		Period (night)	40.20	24.06	14	1.67	0.117
		Season (winter)	−9.48	22.71	14	−0.41	0.683
		Period × Season	−20.56	26.91	14	−0.76	0.458
		sin(hour)	14.69	3.73	1975	3.94	<0.001
		cos(hour)	29.64	9.86	1975	3.01	0.002
		sin(hour) × cos(hour)	55.05	7.64	1975	7.20	<0.001
CO_2_	B	φ	0.93	-	-	-	-
		Intercept	627.76	18.94	1842	33.15	<0.001
		Period (night)	−12.56	33.86	14	−0.37	0.716
		Season (winter)	382.52	70.94	14	5.39	<0.001
		Period × Season	−112.63	80.20	14	−1.40	0.182
		sin(hour)	−15.11	8.57	1842	−1.76	0.078
		cos(hour)	96.02	19.38	1842	4.96	<0.001
		sin(hour) × cos(hour)	−125.92	80.20	14	−5.67	<0.001
NH_3_	A	φ	0.92	-	-	-	-
		Intercept	1.86	0.19	1975	9.77	<0.001
		Period (night)	0.05	0.17	14	0.27	0.790
		Season (winter)	−0.30	0.28	14	−1.06	0.306
		Period × Season	−0.02	0.20	14	−0.09	0.929
		sin(hour)	−0.05	0.03	1975	−1.61	0.108
		cos(hour)	0.25	0.08	1975	3.23	0.001
		sin(hour) × cos(hour)	−0.20	0.06	1975	−3.11	0.002
NH_3_	B	φ	0.97	-	-	-	-
		Intercept	1.99	0.21	1842	9.37	<0.001
		Period (night)	−0.69	0.39	14	−1.79	0.095
		Season (winter)	2.16	0.63	14	3.43	0.004
		Period × Season	1.70	0.95	14	1.79	0.095
		sin(hour)	0.41	0.07	1842	5.97	<0.001
		cos(hour)	0.95	0.16	1842	6.09	<0.001
		sin(hour) × cos(hour)	−0.97	0.18	1842	−5.46	<0.001
PM_2.5_	A	φ	0.90	-	-	-	-
		Intercept	9.21	2.50	1305	3.68	<0.001
		Period (night)	2.27	2.56	14	0.89	<0.001
		Season (winter)	25.37	5.81	14	4.37	<0.001
		Period × Season	1.12	5.52	14	0.20	0.842
		sin(hour)	1.94	0.63	1305	3.08	0.002
		cos(hour)	−2.90	1.06	1305	−2.75	0.006
		sin(hour) × cos(hour)	−4.44	1.15	1305	−3.86	<0.001
PM_2.5_	B	φ	0.67	-	-	-	-
		Intercept	11.28	3.03	1212	3.72	<0.001
		Period (night)	−1.54	2.48	14	−0.62	0.546
		Season (winter)	5.75	4.43	14	1.30	0.215
		Period × Season	−2.36	3.27	14	−0.72	0.482
		sin(hour)	0.64	0.74	1212	0.86	0.390
		cos(hour)	0.24	1.19	1212	0.21	0.838
		sin(hour) × cos(hour)	−1.14	1.14	1212	−0.85	0.393
THI	A	φ	0.99	-	-	-	-
		Intercept	71.8	0.81	1975	88.20	<0.001
		Period (night)	0.12	1.31	14	0.09	0.928
		Season (winter)	−26.07	1.23	14	−21.14	<0.001
		Period × Season	0.41	2.13	14	0.19	0.850
		sin(hour)	−5.24	0.12	1975	−43.15	<0.001
		cos(hour)	−2.23	0.32	1975	−7.05	<0.001
		sin(hour) × cos(hour)	3.62	0.21	1975	17.34	<0.001
THI	B	φ	0.99	-	-	-	-
		Intercept	72.58	1.54	1842	47.09	<0.001
		Period (night)	0.03	1.24	14	0.03	0.979
		Season (winter)	−12.26	2.18	14	−5.63	<0.001
		Period × Season	0.10	1.43	14	0.07	0.943
		sin(hour)	−2.06	0.06	1842	−33.29	0.0000
		cos(hour)	−0.32	0.18	1842	−1.84	0.662
		sin(hour) × cos(hour)	0.72	0.19	1842	3.71	<0.001

**Table 5:** Fixed-effect estimates from the linear mixed-effects models (LMMs) describing the mean concentrations of CO_2_, NH_3_, PM_2.5_, and THI during summer and winter, comparing new and old litter. φ is the temporal autocorrelation coefficient estimated by the model.

Season	Variable	Effect	Estimate	SE	DF	t-Value	*p*
Summer	CO_2_	φ	0.85	-	-	-	-
		(Intercept)	606.97	17.21	1415	35.28	<0.001
		New vs. old litter	−62.81	21.56	8	−2.91	0.0195
		sin(hour)	49.87	9.49	1415	5.26	<0.001
		cos(hour)	73.65	9.05	1415	8.14	<0.001
		sin(hour) × cos(hour)	−15.92	17.27	1415	−0.92	0.357
	NH_3_	φ	0.96	-	-	-	-
		(Intercept)	2.87	0.29	1415	9.76	<0.001
		New vs. old litter	−0.82	0.36	8	−2.30	0.050
		sin(hour)	0.18	0.13	1415	1.45	0.147
		cos(hour)	0.48	0.12	1415	4.13	<0.001
		sin(hour) × cos(hour)	−0.41	0.16	1415	−2.52	0.012
	PM_2.5_	φ	0.92	-	-	-	-
		(Intercept)	11.72	1.84	880	6.37	<0.001
		New vs. old litter	−0.13	2.58	8	−0.05	0.962
		sin(hour)	1.55	0.85	880	1.82	0.069
		cos(hour)	−0.43	0.80	880	−0.54	0.589
		sin(hour) × cos(hour)	−1.82	1.37	880	−1.33	0.183
Winter	CO_2_	φ	0.89	-	-	-	-
		(Intercept)	571.11	11.53	1600	49.53	<0.001
		New vs. old litter	−38.46	14.25	10	−2.70	0.022
		sin(hour)	−12.09	7.46	1600	−1.62	0.105
		cos(hour)	25.50	6.96	1600	3.66	0.0003
		sin(hour) × cos(hour)	−0.19	12.71	1600	−0.02	0.988
	NH_3_	φ	0.85	-	-	-	-
		(Intercept)	2.36	0.16	1600	15.14	<0.001
		New vs. old litter	−0.80	0.22	10	−3.72	0.004
		sin(hour)	−0.02	0.04	1600	−0.45	0.654
		cos(hour)	0.34	0.04	1600	8.20	<0.001
		sin(hour) × cos(hour)	−0.19	0.08	1600	−2.37	0.018
	PM_2.5_	φ	0.92	-	-	-	-
		(Intercept)	18.17	4.39	1061	4.14	<0.001
		New vs. old litter	18.39	6.55	10	2.81	0.019
		sin(hour)	−0.36	2.42	1061	−0.15	0.883
		cos(hour)	2.01	2.22	1061	0.90	0.366
		sin(hour) × cos(hour)	2.20	3.19	1061	0.69	0.490

## Data Availability

The original contributions presented in the study are included in the article; further inquiries can be directed to the corresponding author.
